# A role for LIMA1 in the assembly of actin bundle-supported protrusions

**DOI:** 10.1091/mbc.E26-02-0073

**Published:** 2026-06-10

**Authors:** Jennifer B. Silverman, Kianna L. Robinson, Su Min Hong, Evan S. Krystofiak, Matthew J. Tyska

**Affiliations:** ^a^Department of Cell and Developmental Biology, Vanderbilt University School of Medicine, Nashville, TN 37232; ^b^Vanderbilt Cell Imaging Shared Resource, Vanderbilt University, Nashville, TN 37232; MRC Laboratory of Molecular Biology

## Abstract

Actin bundle-supported membrane protrusions contribute to a range of physiological functions across cell types. An interesting case is the tuft cell, which assembles exaggerated microvillus-like protrusions that support chemosensory functions in the intestinal tract. Tuft cell protrusions are supported by large core actin bundles; several F-actin bundlers are expressed in tuft cells, and these exhibit regionalized localization along the core bundle axis. Uniquely, LIM domain and actin binding 1 (LIMA1) is restricted to the basal, rootlet ends of these bundles. How LIMA1 impacts protrusion formation and core bundle architecture remains unclear. Here, we leveraged multiple forms of microscopy to examine the impact of LIMA1 on the actin cytoskeleton in epithelial and nonepithelial models. Our findings indicate that LIMA1 expression is sufficient to drive the elongation of microvilli in epithelial cells, as well as the formation and stabilization of exaggerated filopodia in a non-epithelial context. Importantly, we found that individual filopodia in LIMA1-overexpressing cells exhibit merging dynamics, which enable the formation of large core bundles from smaller precursors. These data indicate that LIMA1 exerts potent effects on rootlet actin architecture and is well positioned to support the formation and maintenance of large core actin bundles, like those assembled by tuft cells.

## Abstract (executive summary)

- F-actin bundlers are essential for forming the core bundles that support membrane protrusions. Tuft cells build exaggerated core bundles, which are decorated by LIMA1, but how this factor impacts actin architecture is unknown.
- This study leveraged super-resolution microscopy and live imaging to examine the impact of LIMA1 on actin architecture in epithelial and nonepithelial cells. We found that LIMA1 increases protrusion length and drives the formation of large actin bundles via the merging of smaller adjacent protrusions.
- This work provides evidence that LIMA1 plays a role in the formation of the core actin bundles that support microvilli and microvilli-like protrusions.

## INTRODUCTION

Actin bundle-supported membrane protrusions are conserved surface specializations that facilitate a range of physiological functions across multiple classes of epithelial and non-epithelial cell types (Tilney *et al.*, 1980; Ramirez-Weber and Kornberg, 1999; Hsiung *et al.*, 2005; Crawley *et al.*, 2014; Gallop, 2020). Although a micron-scale “core bundle” composed of uniformly polarized, tightly crosslinked actin filaments is a defining feature of these protrusions, the unique functional demands of different tissue contexts are met by variations on this simple structural theme. For example, filopodia, which extend from the leading edge of migrating cells to probe the external environment, are supported by ∼10–30 actin filaments crosslinked by Fascin and vary greatly in length up to 10s of microns depending on cell type (Yamashiro-Matsumura and Matsumura, 1986; Lewis and Bridgman, 1992; Vignjevic *et al.*, 2006a). Cytonemes represent elaborate filopodia that can extend over 100s of microns and are optimized for long-range developmental signaling (Ramirez-Weber and Kornberg, 1999; Hsiung *et al.*, 2005). Stereocilia, which grow from the apex of cochlear and vestibular hair cells to support mechanotransduction, contain hundreds of actin filaments crosslinked by Fascin-2, Plastins-1 and -3, and Espin-1 (Tilney *et al.*, 1989; Zheng *et al.*, 2000; Chou *et al.*, 2011), and can reach lengths in excess of 100 µm (Tilney *et al.*, 1980; Manor and Kachar, 2008). Enterocyte microvilli, which expand apical membrane surface area and maximize intestinal nutrient uptake, are in contrast much shorter at ∼1 µm; these structures are supported by a core of 20–30 actin filaments that are bundled by Villin, Plastin-1, Espin, and MISP (Bretscher and Weber, 1979; Bretscher, 1981; Loomis *et al.*, 2003; Morales *et al.*, 2022). Given the striking diversity in protrusion morphology and function, an important goal is to understand how the unique complement of actin filament bundlers expressed in a specific cellular context gives rise to a distinct protrusion architecture.

F-actin bundling proteins play central roles in shaping unipolar core actin bundles. Most conventional bundlers exhibit multivalent filament binding capacity with two or more actin binding domains (Rajan *et al.*, 2023). Different bundlers link actin filaments at characteristic inter-filament distances, which are dictated by the atomic dimensions of the bundler and the spacing between filament binding domains. For example, Fascin generates tightly packed bundles with ∼8 nm interfilament separation, whereas Villin and Espin generate gaps of ∼12 nm between bundled polymers (Purdy *et al.*, 2007; Hampton *et al.*, 2008; Winkelman *et al.*, 2016). Mixing bundlers with different physical properties impacts filament packing order and bundle rigidity (Krey *et al.*, 2016). Additionally, bundlers promote the elongation of bundles by modulating the kinetics of monomer incorporation or disassembly from constituent filaments (Loomis *et al.*, 2003). Bundlers also impact filament stability and thus length by interfering with the binding of actin-severing proteins (Fattoum *et al.*, 1983). The characteristics of individual actin bundling proteins, including their dimensions, structural properties, and binding affinity for actin filaments, collectively control the local biochemical and mechanical properties of the bundles they create (Loomis *et al.*, 2003; Pollard, 2016; Rajan *et al.*, 2023).

Within epithelial tissues, tuft cells offer a striking example of protrusion assembly that might offer valuable insight into the relationship between actin bundlers and the bundle architectures they produce. Intestinal tuft cells are a rare, chemosensory cell type, responsible for sensing luminal parasites and initiating immune responses (Gerbe *et al.*, 2016; Howitt *et al.*, 2016; von Moltke *et al.*, 2016). Tuft cells are distinguished by an apical “tuft” of ∼100 protrusions, exaggerated microvilli that reach ∼2 µm into the lumen. Remarkably, each protrusion is supported by a long core bundle, comprised of ∼100 hexagonally packed actin filaments, which extends its basal rootlet > 7 µm into the subapical cytoplasm, terminating just above the perinuclear region of the cell. Recent studies from our group revealed that tuft cells express a distinct complement of actin-binding proteins, including phosphorylated Girdin (Y1798), Advillin, Espin, Fimbrin, α-Actinin, and LIM-domain and actin-binding 1 (LIMA1; Esmaeilniakooshkghazi *et al.*, 2020; Hofer and Drenckhahn, 1996; Kuga *et al.*, 2017; Silverman *et al.*, 2024). Super-resolution microscopy also showed that these factors exhibit regionalized localization patterns along the core bundle axis, with most proteins demonstrating enrichment at the apical, membrane-wrapped distal end. In contrast, LIMA1 localization is restricted to the subapical rootlet of the core bundle. Such targeting is reminiscent of mitotic spindle positioning (MISP), an actin bundler that localizes specifically to the rootlets of enterocyte microvilli, where it stabilizes and elongates these structures (Morales *et al.*, 2022).

LIMA1, also known as epithelial protein lost in neoplasm (EPLIN), was initially identified through its downregulation in cancer (Maul and Chang, 1999). LIMA1 is expressed from two major splice forms, LIMA1-α (600 a.a.) and LIMA1-β (759 a.a.), the latter of which includes three additional exons that encode an extended N-terminus (Chen *et al.*, 2000). Both isoforms contain two actin-binding domains that flank a central LIM domain (a.a. 388–488; Maul *et al.*, 2003). The LIMA1 LIM domain lacks the mechanosensitive F-actin binding demonstrated by other LIM domain-containing proteins (Sun *et al.*, 2020). LIMA1 interacts with α-catenin at the junctions of endothelial cells (Chervin-Petinot *et al.*, 2012), and also binds to myosin-5b in the enterocyte (Zhang *et al.*, 2018). In vitro studies of LIMA1 found that it was sufficient to cross-link actin filaments, slow the rate of filament depolymerization, and inhibit the binding of Arp2/3 (Maul *et al.*, 2003). Whether LIMA1 is sufficient to drive the formation of unipolar core actin bundles in cells remains unclear.

Given the enrichment of LIMA1 in tuft cells, its localization to core actin bundle rootlets, and the actin cross-linking and stabilizing roles of LIMA1 revealed in vitro, we hypothesized that LIMA1 is a strong candidate for driving the formation of the large core actin bundles that support tuft protrusions. To test this, we leveraged super-resolution imaging, live-cell imaging, and correlative light and electron microscopy to investigate the impact of LIMA1 on actin architecture in both epithelial and non-epithelial cells. We found that endogenous LIMA1 localizes to the basal ends of multiple actin-based protrusions, including microvilli and filopodia. Moreover, exogenous expression of LIMA1 was sufficient to drive the formation of large actin bundles, resulting in longer microvilli and exaggerated filopodia. Importantly, we found that LIMA1 drove the formation of exaggerated filopodia by promoting the merging of smaller existing protrusions. These findings provide insight into the role of LIMA1 in diverse epithelial contexts and advance our understanding of the cellular mechanisms that drive the formation of core actin bundle-supported protrusions.

## RESULTS

### LIMA1 localizes to core actin bundle rootlets in tuft cells and enterocytes

Previous work from our group established that the core actin bundles found in mouse intestinal tuft cells contain a complement of actin-binding proteins that occupy distinct but overlapping segments along the bundle axis (Silverman *et al.*, 2024). That analysis revealed LIMA1 is unique in this group in that it demonstrates highly specific rootlet localization. To further develop insight into LIMA1 subcellular localization, we used Nikon spatial array confocal (N-SPARC) super-resolution microscopy to image tuft cells in mouse small intestinal sections (Figure 1A). Consistent with our earlier studies, reconstructed image volumes revealed that the LIMA1 signal was strongly enriched on rootlets, appearing in bright puncta that were well aligned along core bundles. Because previous work established that mouse and human tuft cells exhibit moderate heterogeneity in cytoskeletal protein expression (Banerjee *et al.*, 2020), we next sought to determine if LIMA1 demonstrated similar rootlet localization in human intestinal tuft cells. Super-resolution imaging of human samples revealed that, as in mouse tuft cells, LIMA1 was restricted to core bundle rootlets (Figure 1B). Although the formalin fixation and paraffin-embedding (FFPE) used to prepare these samples limited our ability to observe fine structure, we did note that the LIMA1 signal began ∼0.5 µm below the apical surface and was highest on more basal segments of core bundles. Surprisingly, these images also revealed that human LIMA1 was expressed in enterocytes, where it was localized to the rootlets of brush border microvilli (Figure 1B, yellow arrow). In mouse enterocytes, signal on microvillar rootlets was barely detectable (Figure 1A, yellow arrow), although perceived differences in mouse vs. human expression and localization could be explained by our choice of antibody (anti-human LIMA1) and fixation method, as FFPE mouse tissue showed similar LIMA1 localization to microvillar rootlets (Supplemental Figure S1). Nevertheless, these results indicate that LIMA1 holds a strong preference for targeting core bundle rootlets, independent of the specific epithelial cell context.

**FIGURE 1:**
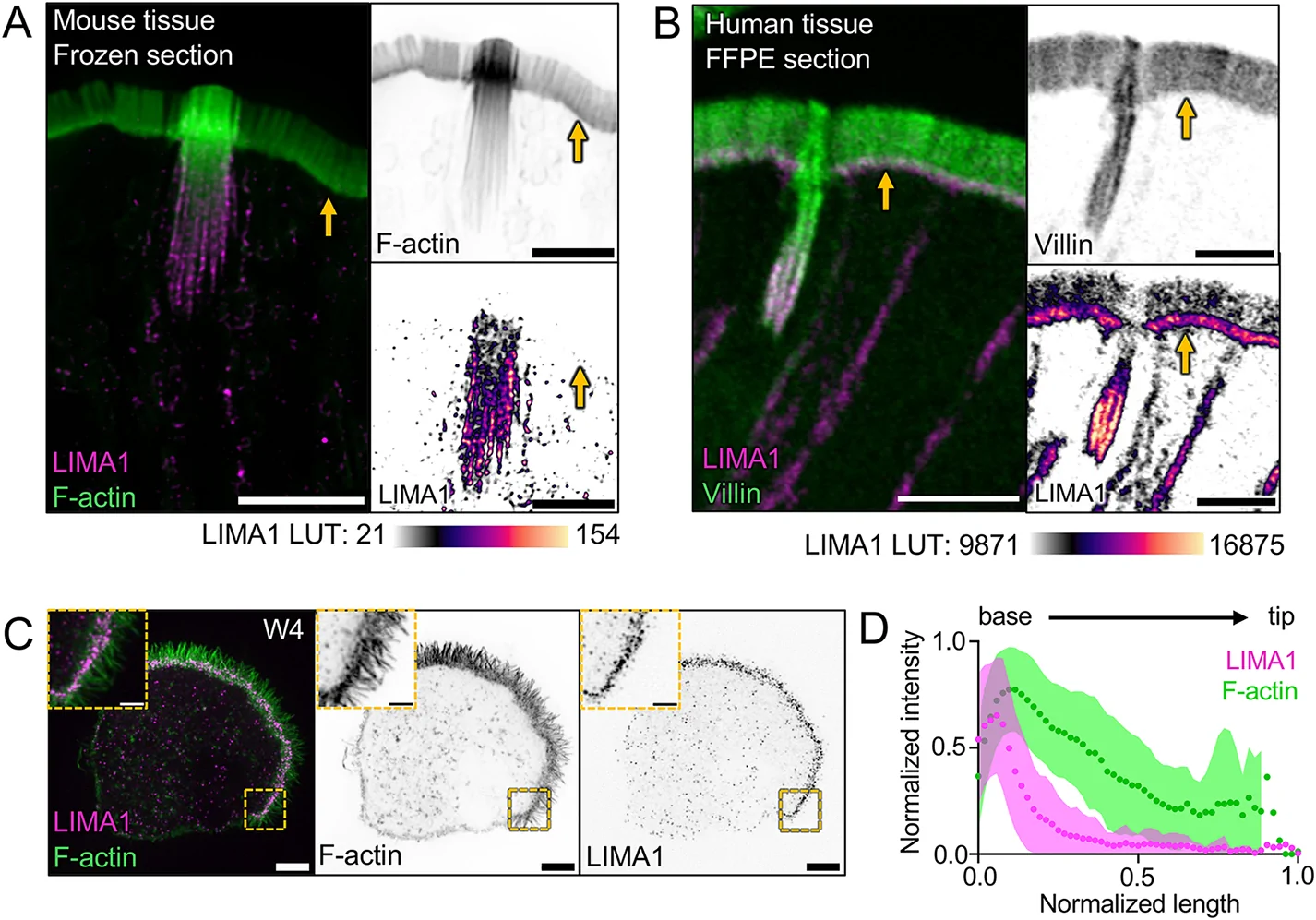
LIMA1 localizes to the rootlets of epithelial actin-based protrusions. A) MaxIP of an NSPARC image volume showing a mouse intestinal tissue frozen section stained with phalloidin to label F-actin (merge) and anti-LIMA1 (magenta); *right*, single channel images, LIMA1 signal intensity is shown with an intensity-scaled LUT. Scale bar = 5 µm. Yellow arrows indicate microvillar rootlets. B) Single plane Airyscan image of a formalin fixed paraffin embedded (FFPE) human intestinal tissue section stained with anti-villin to label F-actin (villin) and anti-LIMA1 (magenta); *right*, single channel images, LIMA1 signal intensity is shown with an intensity-scaled LUT. Scale bar = 5 µm. C) MaxIP of an SDC image volume showing a polarized W4 cell stained with phalloidin to label F-actin (green) and anti-LIMA1 (magenta); *right*, inverted single channel images. Scale bar = 2 µm. D) Line scans drawn along individual W4 microvilli measuring phalloidin intensity (green) and LIMA1 intensity (magenta). SD is shown as a transparent color band surrounding the mean at each position (circles). *N* = 5 microvilli measured per cell, 31 cells total, sampled from three replicate experiments.

We next sought to determine whether human intestinal epithelial cell lines recapitulate the strong rootlet localization we observed in native tissues. To this end, we examined LIMA1 immunostaining in Ls174T-W4 (W4) cells, an intestinal epithelial cell line that can be induced to polarize and form apical microvilli (Baas *et al.*, 2004). Confocal imaging of these samples revealed that LIMA1 was enriched at the base of microvilli, in a punctate manner reminiscent of mouse tuft cell rootlets (Figure 1, C and D). Thus, LIMA1 preferentially binds core actin bundle rootlets in tuft cells and enterocytes from native mouse and human intestinal tissues, and in the W4 human cell culture model.

### LIMA1 overexpression increases microvillar length

Tuft cells express higher levels of LIMA1 relative to neighboring enterocytes (Silverman *et al.*, 2024), and this molecular distinction might explain the striking differences in protrusion architecture between these cell types. From this perspective, we sought to determine if supplementing epithelial cells with LIMA1 resulted in the generation of larger core actin bundles. We generated an mScarlet3 (mS3) tagged variant of human LIMA1-β (LIMA1), expressed this construct in W4 cells, and used confocal microscopy to assess potential impacts on cell morphology. Overexpressed LIMA1 was highly enriched on microvillar rootlets, although this signal also extended distally along the microvillar shaft (Figure 2, A–C). Measurements of microvilli length revealed that LIMA1 overexpression induced a modest, but significant elongation (control: 3.0 ± 0.6 µm; mS3-LIMA1: 3.5 µm ± 0.5; Figure 2D). Unexpectedly, LIMA1 overexpression also impacted the position of microvilli on the cell surface. W4 cells typically extend apical microvilli laterally upon induction of polarization. However, LIMA1 overexpression markedly increased the percentage of cells that produced microvilli dorsally, rather than laterally (Figure 2, E and F). Cells with mispositioned brush borders also assembled large, surface-associated filopodia that were rich in LIMA1 (Figure 2E). To determine whether the effects of LIMA1 were specific to the intestinal epithelial context, we overexpressed LIMA1 in LLC-PK1-CL4 (CL4) cells, a monolayer-forming pig kidney epithelial cell line that builds apical microvilli. Similar to experiments in W4 cells, overexpressed LIMA1 targeted to rootlets and significantly increased microvilli length (control: 1.6 ± 0.3 µm; mS3-LIMA1: 2.8 ± 0.9 µm; Figure 2, G–I). Together, these data indicate that supplementing cells with higher levels of LIMA1 promotes core bundle elongation, a finding that is generally consistent with the behavior of other bundling proteins (e.g., Espin and MISP; Loomis *et al.*, 2003; Morales *et al.*, 2022).

**FIGURE 2:**
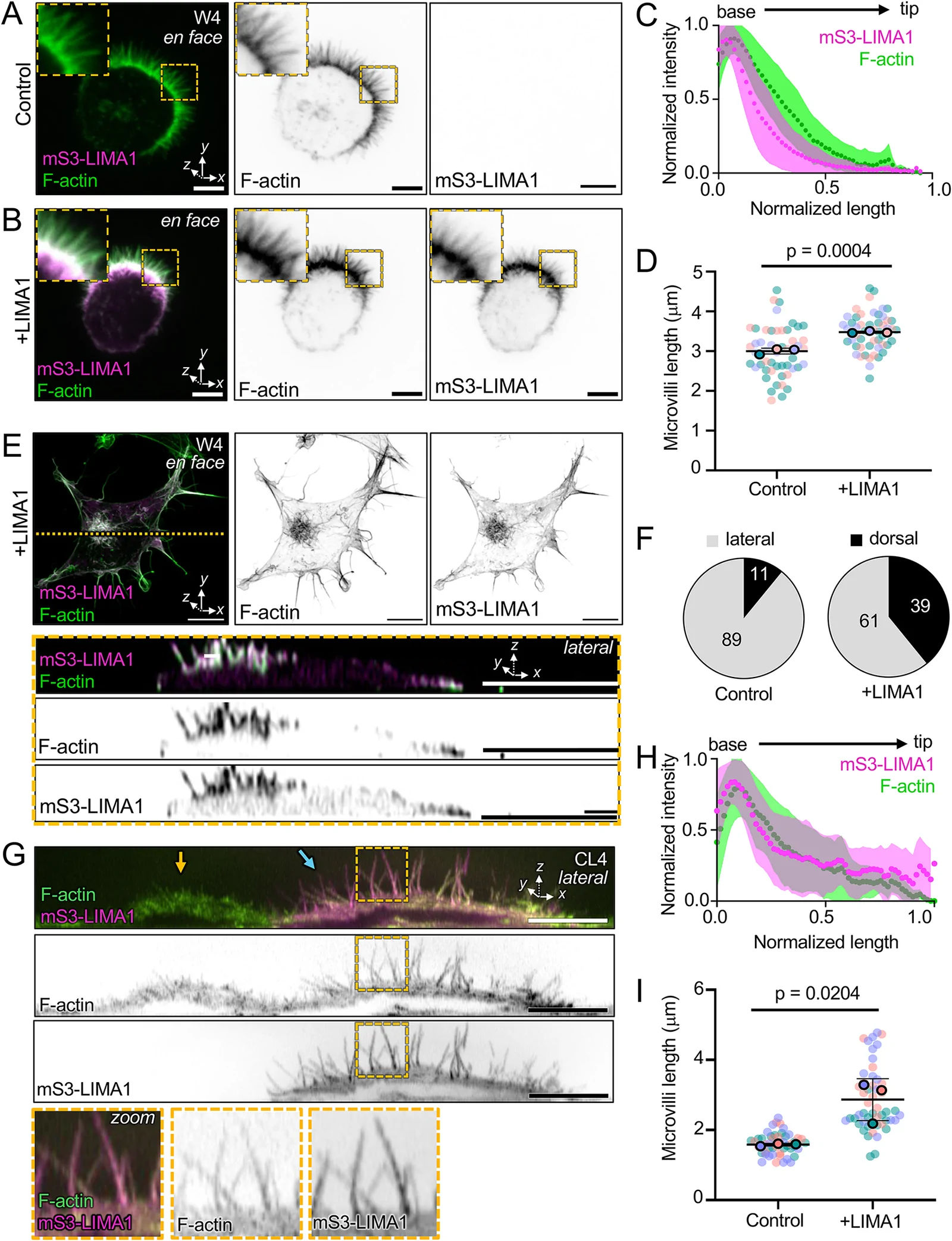
Overexpression of LIMA1 induces longer microvilli in epithelial cell culture models. A) MaxIP of SDC image volume showing a non-expressing control W4 cell stained with phalloidin to label F-actin (green). B) MaxIP of SDC volume of a W4 cell overexpressing mS3-LIMA1 (magenta) and stained with phalloidin to label F-actin (green). Scale bars for A and B = 5 µm. C) Line scans drawn along individual microvilli to measure phalloidin (green) and LIMA1 intensity (magenta). Mean intensities (circles) are shown with the transparent color band representing SD; *N* = 200 microvilli, from 51 cells sampled from three replicate experiments. D) W4 cell microvillar length measurements; *N* = 5 microvilli per cell, 51 cells sampled from three replicate experiments in LIMA1 overexpressing versus control cells. Unpaired *t* test, *p* value is shown on the plot. E) *top*, MaxIP of an SDC image volume showing a W4 cell overexpressing mS3-LIMA1; *bottom*, a single lateral slice from the same cell taken along the yellow dotted line (below). F) Percentage of polarized W4 cells containing dorsally or laterally oriented microvilli in control and LIMA1 OEx cells; *N* = 258 cells from three replicate experiments. G) MaxIP of SDC image of CL4 cells overexpressing mS3-LIMA1 (magenta) and stained for phalloidin to label F-actin (green); view shows a lateral MaxIP of three z slices. Scale bar = 5 µm. H) Linescans drawn along individual microvilli in the lateral view to measure phalloidin intensity (green) and LIMA1 intensity (magenta). Mean intensities (circles) are shown with the transparent color band representing SD. *N* = 3 microvilli measured per cell; 29 cells total sampled from three replicate experiments. I) Microvillar length measurements from LIMA1 overexpressing and control CL4 cells; *N* = 4–10 microvilli measured per cell, 100 cells from three replicate experiments. Unpaired *t* test, *p* value shown on plot.

### LIMA1 is sufficient to drive the formation of filopodia in HeLa cells

While LIMA1 supplementation was sufficient to increase the length of microvilli in W4 and CL4 cells (alluded to above), we did not observe significant effects on rootlet structure. Phenotypes in these epithelial models could potentially be muted by competition with the multiple strong actin bundling proteins expressed in these cells, including Espin, Fimbrin, Villin, and MISP (Bretscher and Weber, 1979; Bretscher, 1981; Loomis *et al.*, 2003; Morales *et al.*, 2022). To further explore this possibility, we examined LIMA1 localization and overexpression phenotypes in the context of HeLa cells, a nonepithelial culture model that lacks apical-basal polarity. Antibody staining and confocal microscopy revealed that endogenous LIMA1 was found throughout the cell on a range of actin-rich structures. However, we also noted strong localization to the base of filopodia (Figure 3, A and B), which, like microvilli, are supported by unipolar core bundles of actin filaments. Remarkably, LIMA1 overexpression in HeLa cells induced the formation of numerous exaggerated protrusions, which appeared longer and thicker compared with those extending from control cells; a similar exaggerated phenotype was also observed in mouse B16 melanoma cells (Supplemental Figure S2). Examination of phalloidin levels in these protrusions using an intensity-coded LUT suggested higher intensities (Figure 3, C and D). Analogous to our results from epithelial cell culture models, exogenous LIMA1 in HeLa cells localized most prominently to the basal ends of filopodial core bundles (Figure 3E). To determine whether the protrusions formed by LIMA1 overexpressing cells represent canonical filopodia, we stained for established filopodial residents Fascin (Figure 3F) and VASP (Figure 3G) and found a strong signal for both factors in induced protrusions. Together, these data indicate that LIMA1 expression in non-epithelial cells is sufficient to remodel the actin cytoskeleton and drive the formation of exaggerated filopodia.

**FIGURE 3:**
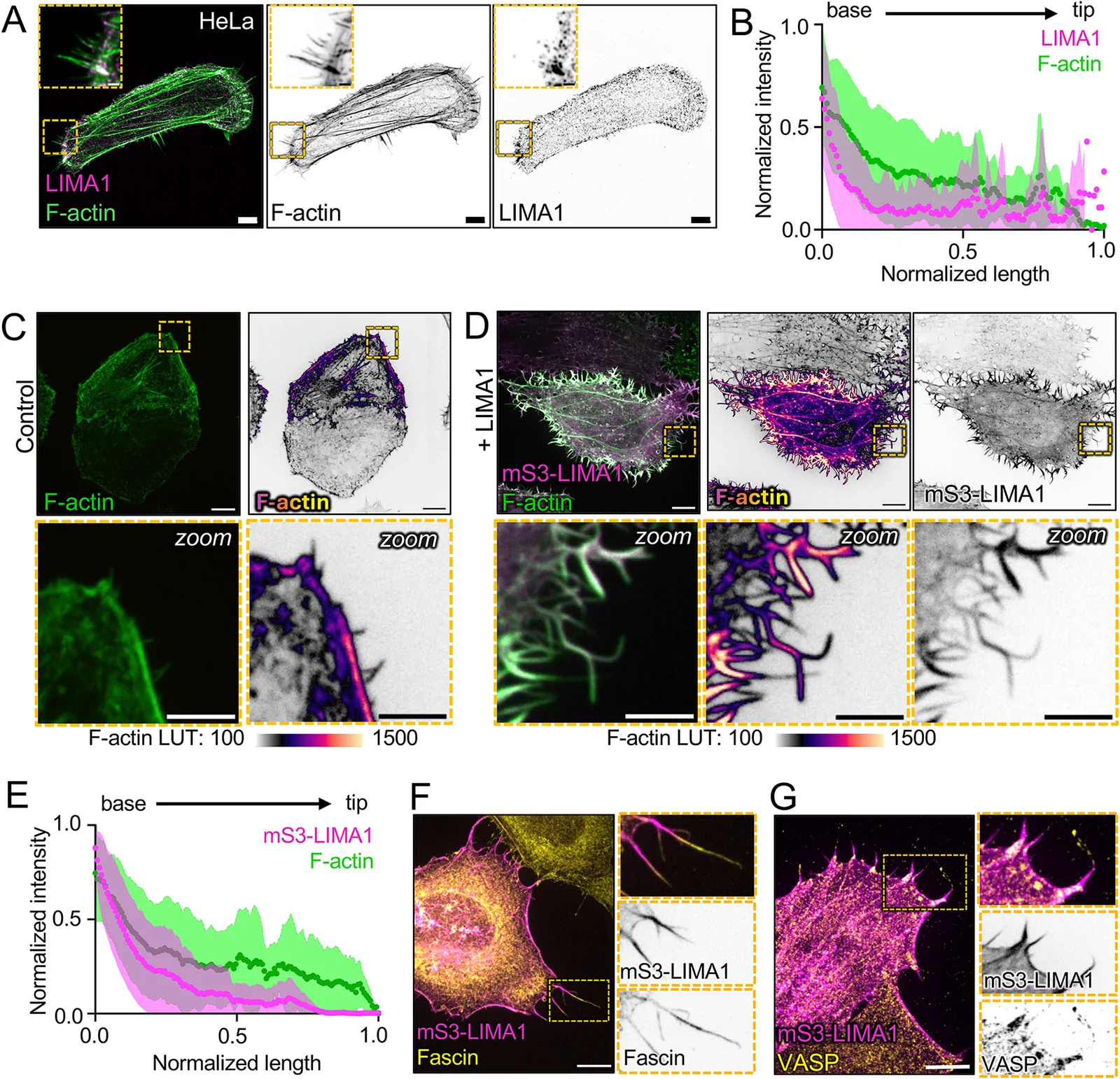
LIMA1 overexpression drives the formation of exaggerated filopodia in HeLa cells. A) MaxIP of a SDC image volume showing a HeLa cell stained with anti-LIMA1 (magenta) and phalloidin to label F-actin (green). Scale bar = 5 µm. B) Line scans drawn along individual filopodia to measure phalloidin (green) and LIMA1 intensity (magenta). Mean intensities (circles) are shown with the transparent color band representing SD. Five filopodia were measured per cell, 30 cells total sampled over 3 replicates. C) and D) MaxIP of a SDC image volume showing control (C) and mS3-LIMA1 overexpressing HeLa cells (D); mS3-LIMA1 shown in magenta and F-actin in green. Scale bar = 10 µm, zoom view scale bars = 5 µm. Single phalloidin channel marked with intensity-scaled LUT for both C and D. E) Linescan analysis of mS3-LIMA1 along filopodia. Line scans drawn along individual filopodia to measure phalloidin (green) and LIMA1 intensity (magenta). Mean intensities (circles) are shown with the transparent color band representing SD. *N* = 5 filopodia measured per cell, 32 cells total sampled over three replicate experiments. F and G) MaxIP of an SDC image volume showing mS3-LIMA1 (magenta) overexpressing cells stained with anti-fascin (F) and anti-VASP (G), both shown in yellow. Scale bar = 5 µm.

### LIMA1 increases the number and length of filopodia

To further characterize the filopodia assembled by LIMA1 overexpressing cells, we leveraged an endogenously tagged (endo-) mStayGold-epidermal growth factor receptor pathway substrate 8 (EPS8) HeLa cell line created using CRISPR/Cas9 genome editing. EPS8 is a barbed-end binding protein found at the distal tips of protrusions supported by unipolar actin bundles, including microvilli, stereocilia, and filopodia (Roffers-Agarwal *et al.*, 2005; Manor *et al.*, 2011; Gaeta *et al.*, 2021). In endo-EPS8 HeLa cells, filopodia were decorated with distal tip puncta as expected (Figure 4A). Moreover, the endogenous EPS8 signal displayed the same localization pattern as overexpressed mStayGold-EPS8 (Supplemental Figure S3). In LIMA1 overexpressing cells, EPS8 was found at the tips of all visible filopodia, including the tips of bifurcated protrusions, suggesting that actin filaments in these structures are uniformly polarized with a “barbed end out” orientation (Figure 4B). To determine if LIMA1 overexpression increased the number of filopodia, we measured the number of distal tip-associated EPS8 puncta per micron perimeter of cell body; this analysis revealed a significant increase in the number of puncta compared with controls (control: 0.7 ± 0.3; mS3-LIMA1: 1.4 ± 0.8) (Figure 4C). Interestingly, the intensity of endo-EPS8 in distal tip puncta also trended upwards in LIMA1 overexpressing cells (control: 166.2 ± 16.2; mS3-LIMA1: 174.2 ± 18.3), potentially indicating an increased number of barbed ends at filopodial tips (Figure 4D). Analysis of the spatial distribution of EPS8 puncta revealed that they were further from the cell edge in LIMA1 overexpressing cells, suggestive of longer filopodia (control: 0.67 ± 0.3 µm; mS3-LIMA1: 1.9 ± 0.6 µm; Figure 4E). Indeed, manual tracing of individual protrusions revealed that LIMA1 overexpression significantly elongated filopodia (control: 1.6 ± 0.7 µm; mS3-LIMA1: 4.3 ± 2.0 µm; Figure 4F). Moreover, area under the curve (AUC) analysis (Figure 4G) of the phalloidin signal in induced filopodia trended higher than controls (control: 304.9 ± 117.8; mS3-LIMA1: 431.6 ± 172.6; Figure 4H), again suggesting that LIMA1 overexpression may increase the number of filaments per core bundle. Collectively, these data indicate that LIMA1 increases the number, length, and potentially the F-actin content of filopodial core bundles.

**FIGURE 4:**
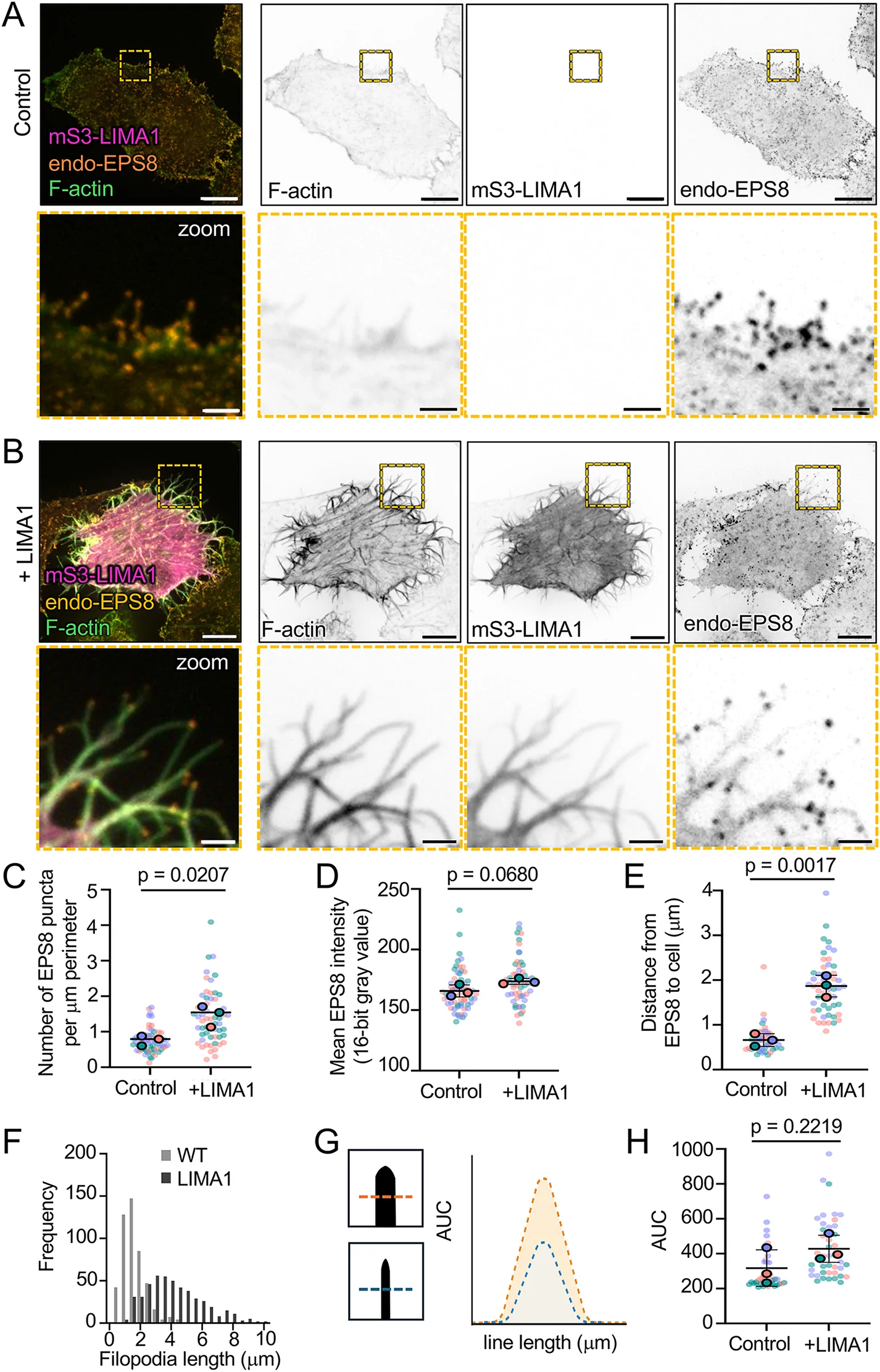
LIMA1 overexpressing HeLa cells build large numbers of long filopodia. A) and B) MaxIP of an SDC confocal volume showing control HeLa cells expressing endogenously tagged mStayGold-EPS8 (endo-EPS8) alone (A), or with overexpression mS3-LIMA1 (B); endo-EPS8 shown in orange, mS3-LIMA1 in magenta, and phalloidin in green. Scale bar = 10 µm, scale bar in zoom inset = 1 µm. C) Number of filopodial EPS8 puncta per micron cell body perimeter measured for control and LIMA1 OEx cells. *N* = 125 cells measured from three replicate experiments. Unpaired *t* test, *p* value is shown on the plot. D) Mean 16-bit gray value intensity of filopodial EPS8 tip puncta in control versus LIMA1 OEx HeLa cells. *N* = 125 cells quantified from three replicate experiments. Unpaired *t* test, *p* value is shown on the plot. E) Analysis of measurements of the straight-line distance between filopodial EPS8 puncta and the cell body in control versus LIMA1 OEx cells. *N* = 97 cells quantified over three replicates. Unpaired *t* test, *p* value is shown on the plot. F) Histogram showing the lengths of individual filopodia from tip to base for control versus LIMA1 OEx cells. *N* = 958 filopodia measured over 100 cells from three replicate experiments. G) Cartoon depicting area under the curve (AUC) analysis. H) AUC analysis measures phalloidin intensity derived from lines drawn orthogonally at the midsection of filopodia in control or LIMA1 OEx cells. *N* = 4–13 filopodia measured in 43 cells from three replicate experiments. Unpaired *t* test, *p* value is shown on the plot.

### LIMA1-induced filopodia exhibit dynamic merging behavior

We noted that most of the filopodia induced by LIMA1 overexpression exhibited striking bifurcations (Figure 4B) that, based on our experience, are rarely observed under normal conditions. Bifurcations could form if filopodia split at their distal ends, merge at their basal ends, or grow new filopodia laterally from their shafts. To investigate these possibilities, we performed live imaging of endo-EPS8 HeLa cells overexpressing LIMA1. Time-lapse spinning disk confocal (SDC) imaging revealed that filopodia in LIMA1 OEx cells exhibited lateral motion and frequently merged to form a single, larger filopodium, by “zippering” from the base up to the tip (Figure 5A). Interestingly, EPS8 puncta at the tip of the shorter merging filopodium dissociated and appeared to spread along the shaft of the merging core bundles (Figure 5A, arrow). While merging behavior was most common, we occasionally observed bifurcations originating from the growth of a new daughter filopodium from the shaft of an existing protrusion (Figure 5B, arrow). To examine the plasma membrane morphology of bifurcated protrusions induced by LIMA1 overexpression, we labeled cells with a plasma membrane dye (CellBrite) and visualized merging events. These time-lapse studies showed that as the zippering of adjacent filopodia begins, membrane wrapping closely follows the morphology of the merging core bundles, starting at the base and propagating tip-ward until both filopodia coalesce under a single membrane (Figure 5C). To further characterize the morphology of induced filopodia, we used correlative light electron microscopy (EM) to identify EGFP-LIMA1-expressing cells and visualize their surfaces using scanning EM. These images revealed clear examples of bifurcated filopodia and confirmed membrane wrapping and instances of webbing between forks (Figure 5D, arrow). Together, these time-lapse observations reveal that LIMA1 overexpressing cells assemble aberrantly large filopodia through the merging and consolidation of smaller pre-existing protrusions.

**FIGURE 5:**
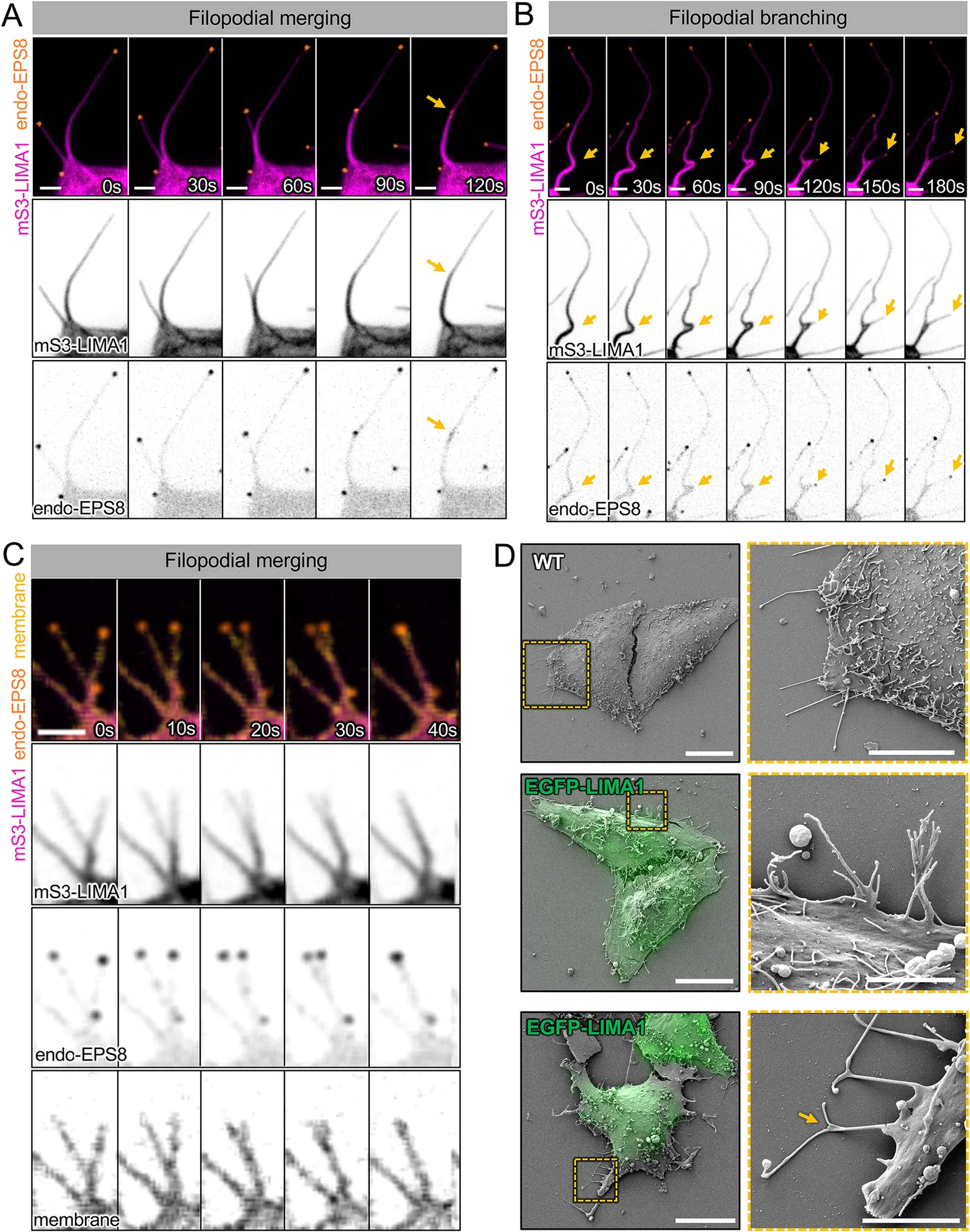
Filopodia in LIMA1 overexpressing Hela cells dynamically merge and branch. A) and B) Single image plane montage created from an SDC timelapse showing endo-EPS8 (orange) HeLa cells with mS3-LIMA1 OEx (magenta) in media supplemented with 0.25% methylcellulose. Arrows indicate completed merge (A) or branching (B) events. Scale bar = 2 µm. C) Single image plane montage created from an SDC timelapse in endo-EPS8 (orange) HeLa cells with mS3-LIMA1 OEx (magenta) and labeled with CellBrite membrane dye (yellow). Scale bar = 2 µm. D) Correlative light and scanning electron microscopy on WT HeLa cells (scale bar = 20 µm, zoom = 10 µm) and EGFP-LIMA1 OEx cells (Scale bar = 20 µm, zoom = 5 µm). The yellow arrow indicates branching in filopodia.

### LIMA1 expression increases filopodial lifetime

To better understand how LIMA1 promotes the formation of exaggerated filopodia, we examined protrusion growth dynamics in LIMA1 overexpressing cells (Figure 6A). To dampen lateral movements and enable tracking of filopodial growth, 0.25% methylcellulose was added before imaging. Time-lapse SDC images revealed that LIMA1-induced filopodia grew slowly and processively, relative to the short-lived filopodia that grew from the surface of control cells. To quantify filopodial motion in more detail, we used the FIJI TrackMate plugin to track individual EPS8 puncta at the tips of large numbers of filopodia (Figure 6B). LIMA1-induced filopodia exhibited significantly longer lifetimes (control: 133.2 ± 60.5 s; mS3-LIMA1: 205.7 ± 33.4 s; Figure 6C) and traveled further than control protrusions (Figure 6D; control: 3.2 ± 1.9 µm; mS3-LIMA1: 4.6 ± 2.0 µm). Using kymograph analysis, we also found that rates of growth and retraction trended slower than those in control cells (control growth: 14 ± 4 nm/s; control retraction: 15 ± 3 nm/s; mS3-LIMA1 growth: 10 ± 3 nm/s; mS3-LIMA1 retraction: 9 ± 4 nm/s; Figure 6, E and F). Finally, photobleaching-based measurements of filopodial retrograde actin flow in cells expressing mNeonGreen-β-actin revealed that treadmilling rates were not significantly impacted by LIMA1 overexpression (control: 11 ± 7 nm/s; mS3-LIMA1: 8 ± 6 nm/s; Figure 6, G and H). Together, these measurements indicate that LIMA1 exerts subtle effects on core bundle growth and retraction dynamics, but a significant impact on protrusion survival, indicated by a > 50% increase in lifetime. This effect might provide a kinetic basis for the accumulation of large numbers of filopodia on the surface of LIMA1 overexpressing cells.

**FIGURE 6:**
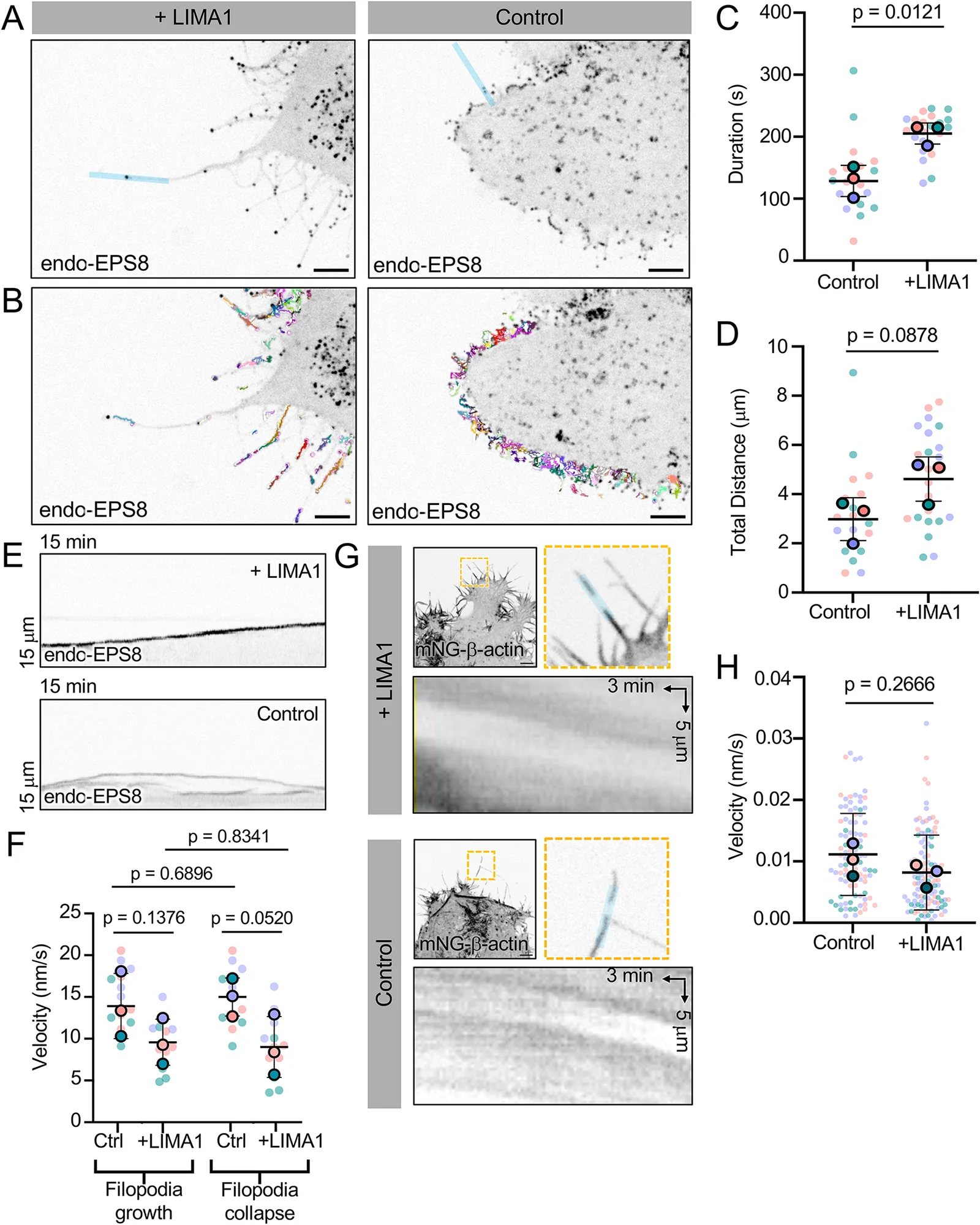
Filopodia extending from LIMA1 overexpressing cells exhibit longer lifetimes. A) Single z-slice of the first frame from an SDC timelapse showing endo-EPS8 control and mS3-LIMA1 OEx HeLa cells in media supplemented with 0.5% methylcellulose. Scale bar = 5 µm. Blue-highlighted lines correspond to kymographs in (E). B) Representative examples of trajectories derived from filopodial EPS8 puncta tracking from the cells shown in (A). C) and D) Duration of individual EPS8 puncta (C) and total distance traveled (D) derived from TrackMate analysis on control and mS3-LIMA1 OEx HeLa cells. *N* = 19 control, 22 LIMA1 OEx cells sampled over three replicates. Unpaired *t* test, *p* values are shown on the plots. E) Kymographs derived from blue lines in (A) of endo-EPS8 puncta in control and mS3-LIMA1 OEx HeLa cells. Box height = 15 µm, box width = 15 min. F) Kymograph-derived velocities of filopodia growth and collapse in control versus mS3-LIMA1 OEx HeLa cells. Two-way ANOVA with multiple comparisons, *p* values are shown on the plot; *N* = 24 cells from three replicates. G) Cells overexpressing mNG-β-Actin alone or mNG-β-actin and mS3-LIMA1. Small regions along the length of filopodia were photobleached and used as markers to visualize actin treadmilling. Kymographs were drawn along the length of the highlighted blue region. H) Velocities of actin treadmilling as determined from kymographs described in (G). *N* = 3–10 filopodia were quantified from 46 cells, from three replicate experiments. Unpaired *t* test, *p* value is shown on the plot.

### LIMA1 exhibits robust turnover in core bundles

To further examine the nature of LIMA1 turnover within filopodia, we performed fluorescence recovery after photobleaching (FRAP) analysis of EGFP-LIMA1 (Figure 7, A and E; Supplemental Table S1). Although LIMA1 fluorescence recovered quickly with a signal that plateaued in less than two minutes (t_1/2_ = 23.1 s), the immobile fraction (1 – mobile fraction) remained large at 0.47. To generate points of comparison, we also performed FRAP analysis on other microvillar actin bundling proteins, including EGFP-Espin, EGFP-Fimbrin, and EGFP-MISP (Figure 7, B–D; Loomis *et al.*, 2003; Morales *et al.*, 2022). Of these bundlers, Espin exhibited the highest immobile fraction at 0.78, while Fimbrin had the lowest at 0.07 (Figure 7, F and G). Interestingly, MISP, which is also found on the rootlets of microvilli, demonstrated a t_1/2_ = 21.5 s and an intermediate immobile fraction at 0.65 (Figure 7H), making it most similar to LIMA1. The range of mobilities measured in these FRAP experiments most likely reflects kinetic differences in the actin binding and bundling properties of each bundler, although we cannot rule out the contribution of other protein:protein or protein:membrane interactions. Nevertheless, a large fraction of the filopodial LIMA1 population (0.53) does turn over on a timescale of seconds, and this may be important for the filopodial remodeling induced by LIMA1 overexpression, as alluded to above.

**FIGURE 7:**
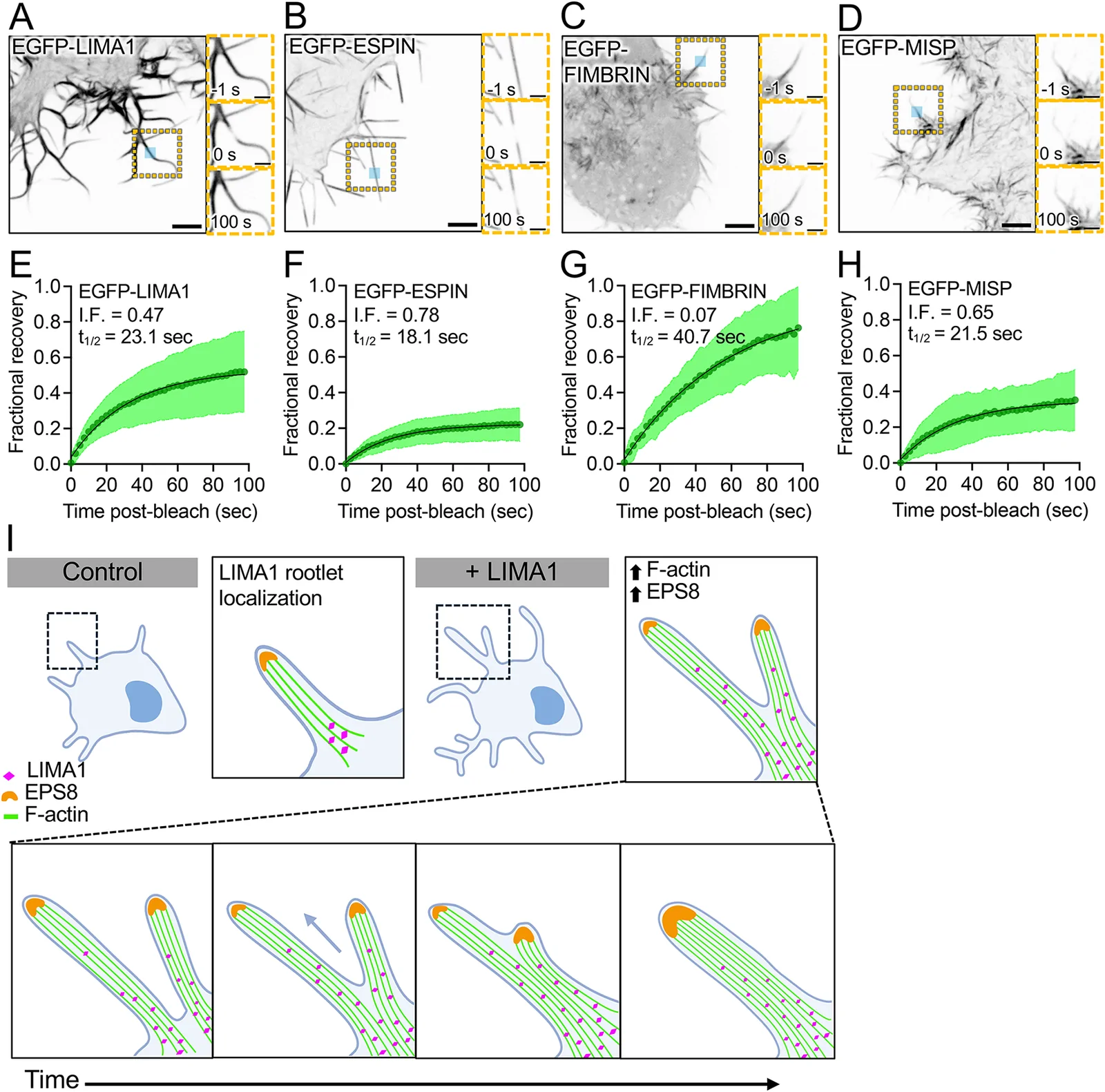
LIMA1 exhibits robust turnover in filopodia. A) and D) Single z-slice SDC timelapse montages of FRAP analysis on HeLa cells expressing EGFP-LIMA1 (A), EGFP-ESPIN (B), EGFP-FIMBRIN (C), or EGFP-MISP (D). The yellow dashed box indicates the zoom shown to the right, and the blue highlighted box indicates the bleached ROI. E)–H) Recovery curves for EGFP-LIMA1 (E), EGFP-Espin (E), EGFP-Fimbrin (G), and EGFP-MISP (H). ROI signals were recorded for 100 s post-bleach; normalized recovery time courses were fit with a one-phase association model to derive immobile fractions (i.e., 1-plateau) and t_1/2_ values, which are shown on each plot and listed in Supplemental Table S1. Scale bars = 2 µm. Zoom inset scale bars = 1 µm. I) Cartoon model of LIMA1-driven filopodial remodeling and bundle expansion.

## DISCUSSION

Although actin bundling proteins are well studied in the context of apical specializations such as the enterocyte brush border and the hair cell hair bundle, how the specific complement of actin-binding proteins expressed by intestinal tuft cells contributes to the unique architecture and function of tuft core bundles remains unclear. Given that our initial cell biological studies of the tuft revealed that LIMA1 is enriched and exhibits highly specific localization to the rootlets of core bundles (Silverman *et al.*, 2024), we sought to understand how this factor impacts actin architecture. While previous studies on LIMA1 demonstrated F-actin crosslinking potential and suggested a role in antagonizing the Arp2/3 branched nucleation complex (Maul *et al.*, 2003), whether LIMA1 is capable of driving the formation of unipolar core actin bundles remained unclear. To fill this gap, we leveraged cell culture and overexpression models to develop our understanding of how LIMA1 impacts the actin cytoskeleton. Below, we discuss our observations and their implications for understanding how epithelial cells assemble large core actin bundles for supporting membrane protrusions.

### LIMA1 targets specifically to the basal ends of unipolar core actin bundles

Our staining experiments revealed that endogenous LIMA1 localizes to the rootlets of tuft cell core actin bundles and enterocyte microvilli, in both mouse and human tissues. This localization was recapitulated in intestinal and kidney cell culture models. Importantly, this pattern of targeting was not restricted to core bundles assembled by epithelial cells, as LIMA1 demonstrated similar enrichment at the basal ends of filopodial core bundles in HeLa cells. Although overexpressed LIMA1 did extend more distally along the core bundle axis, even in highly expressing cells, LIMA1 remained most prominently enriched at the base. Why LIMA1 localizes specifically to the basal/rootlet ends of core bundles remains unknown, although such localization is reminiscent of MISP, an actin bundling protein expressed at high levels in enterocytes where it targets microvillar rootlets (Morales *et al.*, 2022). The association of MISP to rootlet actin filaments is driven in part by antagonism with Ezrin, an actin-membrane linking protein that prevents MISP from binding in the distal, membrane-wrapped portion of the microvillus (Morales *et al.*, 2022). Additionally, in vitro studies with purified MISP revealed a binding preference for “aged” actin filaments, which are enriched in ADP-actin versus ADP-Pi or ATP-actin (Morales *et al.*, 2024). Such a preference might contribute to MISP targeting near filament pointed ends, which are focused in the rootlet segment of the microvillus. Beyond these possible mechanisms, LIMA1 rootlet localization might also be the product of competition with other actin bundling proteins. Espin, for example, is strongly localized to the distal portion of core actin bundles along with Villin, in microvilli, and Advillin in tuft cells, and may prevent LIMA1 from occupying these regions (Silverman *et al.*, 2024). Although Espin and LIMA1 exhibit comparable binding affinities for actin (K_D_ of ∼0.1 µM; Loomis *et al.*, 2003; Maul *et al.*, 2003), Espin might have a kinetic advantage over LIMA1 (e.g., a slower detachment rate), which would favor occupancy in the distal segment of the core bundle. This would be consistent with our FRAP analysis, which showed that Espin exhibits a much larger immobile fraction relative to LIMA1. Competition between these two factors could also be related to the structural details of their F-actin binding interfaces. Further studies with LIMA1 are needed to determine if this factor preferentially binds ADP-actin and/or whether its localization is the product of competition for binding sites with other actin-binding proteins.

### Impact of LIMA1 on protrusion assembly

LIMA1 overexpression in epithelial cell culture models produced only modest structural phenotypes, increasing microvilli length by less than 1 µm. The subtlety of these effects could be due to competition with the large complement of actin bundling proteins expressed in epithelial cells, including Fimbrin, Villin, Espin, and MISP (Bretscher and Weber, 1979; Bretscher, 1981; Morales *et al.*, 2022; Loomis *et al.*, 2003). In contrast to our observations in polarizing epithelial cells, LIMA1 overexpression in HeLa cells was sufficient to drive the formation of highly exaggerated filopodia, which were increased in their numbers, length, and F-actin intensity. These effects are well aligned with previous in vitro studies showing that LIMA1 crosslinks and stabilizes actin filaments, while preventing Arp2/3 binding (Maul *et al.*, 2003). Our findings are also consistent with the expected effects of filament bundler overexpression (Loomis *et al.*, 2003; Vignjevic *et al.*, 2006b; Morales *et al.*, 2024). For example, studies on Fascin collectively indicate that this bundler increases filopodial number, length, and lifetime (Yamashiro *et al.*, 1998; Vignjevic *et al.*, 2006b; Yamakita *et al.*, 2009). Filopodia produced by LIMA1 overexpressing cells were also highly dynamic, demonstrating curling, buckling, and lateral motion, indicating that these bundles are structurally flexible. These dynamic properties suggest that LIMA1 binds to and bundles F-actin while maintaining a short dwell time, which would allow filaments in the core to remain flexible in the bundled state. This point is consistent with our FRAP analysis, which revealed that LIMA1 exhibits intermediate turnover in core bundles when compared with other microvilli-enriched actin-bundling proteins; Espin, which generates long, stable, and straight filopodia in HeLa, exhibited much lower turnover, whereas Fimbrin, which exerts little impact on filopodial structure, demonstrated higher turnover.

### Protrusion merging as a mechanism for assembling large core bundles

Filopodia induced by LIMA1 overexpression exhibited multiple bifurcations along their length. Our live imaging studies revealed that bifurcations most likely represent filopodia captured in the act of merging. In these instances, adjacent protrusions initially make contact and, presumably through the filament bundling activity of LIMA1, zipper up from base to tip, resulting in a larger combined core actin bundle. Interestingly, in vitro studies with purified Fascin reported filament zippering activity during bundle assembly (Sherer and Courtemanche, 2022), suggesting that bundling activity is sufficient to explain these events. Beyond potent filament bundling activity, merging of filopodia might also reflect the dilution of actin-membrane linkers, such as Ezrin or class I myosins (Pelaseyed and Bretscher, 2018). In this scenario, rapid bundle elongation and stabilization caused by LIMA1 overexpression may increase the spacing between membrane-actin linkers, which would lead to a loss of membrane wrapping and, in turn, core bundle consolidation (Tyska *et al.*, 2005; Morales *et al.*, 2022). Merging has been reported previously for short microspikes at the edge of B16F1 cells, which are effectively actin core bundles that lack extensive membrane wrapping (Svitkina *et al.*, 2003). The general idea of remodeling existing actin structures to create core bundles is similar to the “convergent elongation” model, which was originally invoked to explain the Fascin-dependent formation of filopodial core bundles (Svitkina *et al.*, 2003), and more recently as a mechanism that promotes microvilli assembly from clathrin-coated pits (Svitkina *et al.*, 2003; Perkins *et al.*, 2025). Taken together with these previous reports, our findings suggest that consolidation of smaller precursor protrusions could be one mechanism for building core bundles with greater numbers of filaments and thus, larger diameters.

Whether LIMA1 drives the merging of core bundles in the apical domain of differentiating intestinal tuft cells remains unclear. However, previous studies have established that nascent epithelial microvilli are highly dynamic, demonstrating robust motility and collisions (Meenderink *et al.*, 2019). Based on that work, it is tempting to speculate that the exaggerated protrusions comprising the tuft are formed from the consolidation (i.e., merging and fusion) of smaller microvilli core bundles, potentially driven by LIMA1 early in differentiation. A direct test of this proposal would require an epithelial cell culture model that produces tuft cells, which does not yet exist.

Finally, because protrusion dynamics are required for merging-based core bundle growth, other pathways would be needed to expand bundles that maintain a fixed position on the cell surface, such as hair cell stereocilia (Barr-Gillespie, 2015). Indeed, recent studies of hair cell differentiation point to a model where core bundles expand by generating and incorporating short actin filaments at the periphery of the bundle's distal tip; these filaments then elongate down the side of the pre-existing core to increase its diameter (Liao *et al.*, 2025).

### Other functions for LIMA1 in the epithelial core actin bundle

Beyond the clear roles for LIMA1 in promoting core bundle growth and stability, this factor might also contribute to other facets of epithelial cell physiology. For example, core bundles are occupied by a diverse array of actin-binding proteins, and bundlers are well-positioned to control what binds to these structures. LIMA1 targets the nonmembrane wrapped basal/rootlet end of the bundle, which in the case of tuft cells extends several microns into the subapical cytoplasm. Given the unipolar orientation of constituent filaments, these bundles could serve as tracks to support vesicle trafficking throughout this region, along the apical-basal axis. This would be consistent with our recent ultrastructural studies of tuft core bundles, which showed vesicles, including multivesicular bodies, bound directly to rootlets (Silverman *et al.*, 2024). That work also revealed strong tuft cell staining for the processive barbed- and pointed-end directed motors, myosin-5B and myosin-6, respectively, which could both function as transporters in this context (Engevik and Engevik, 2022). In addition to the structural role of bundling filaments together, LIMA1 is well positioned to directly impact the binding of these and other proteins to the rootlets. This possibility is supported by early biochemical studies showing that LIMA1 inhibits the nucleation potential of Arpr2/3 (Maul *et al.*, 2003), potentially by competing for binding sites along the filament. Other investigations suggest that LIMA1 holds important roles outside the context of core actin bundles. Zhang and colleagues demonstrated that LIMA1 directly binds to myosin-5b and can act as a linker to NiemannPick C1-like 1 (NPC1L1), thereby aiding in cholesterol absorption (Zhang *et al.*, 2018). Studies in other cellular contexts suggest that LIMA1 is a binding partner of α-catenin and serves as an indispensable component of the adherens junction (Abe and Takeichi, 2008; Chervin-Petinot *et al.*, 2012). Lastly, several studies have pointed towards different binding partner preferences and roles for LIMA1-α and LIMA1-β isoforms (Taha *et al.*, 2019; Aldirawi *et al.*, 2025; Jantti *et al.*, 2025). Further investigation will be needed to uncover how the effects of LIMA1 on actin architecture and protrusion formation connect to those findings.

## MATERIALS AND METHODS

Request a protocol through *Bio-protocol*

### Experimental model details

#### Mice

Animal experiments were carried out in accordance with Vanderbilt University Medical Center Institutional Animal Care and Use Committee (IACUC) guidelines under protocol #M1600206-02. C57BL6 mice were used for all tissue collections and antibody staining.

#### Cell culture models

HeLa, LLC-PK1-CL4 (CL4), B16F10 (B16) and Ls174T-W4 (W4) cells were cultured at 37°C and 5% CO_2_ in Dulbecco's modified Eagle's medium (DMEM) (Corning #10-013-CV) with high glucose and 2 mM L-Glutamine (Corning #10-0130CV) supplemented with 10% fetal bovine serum (FBS) (R&D Systems) and L-Glutamine (Corning # 25-005-Cl). W4 cell media contained tetracycline-free FBS and was additionally supplemented with 10% tetracycline-free fetal bovine serum (Atlanta Biological, S10350), 1 mg/ml G418 (Gold Biotechnology; G-418), 10 µg/ml blasticidin (Gold Biotechnology; B-800), and 20 µg/ml phleomycin (InvivoGen; ant-ph-1).

## EXPERIMENTAL METHODS

### EGFP-LIMA1 and mS3-LIMA1 construct cloning

Flag-tagged LIMA1β was purchased from Addgene (Plasmid #40956). Standard molecular biological techniques were used to replace the FLAG tag and create an N-terminally tagged EGFP-LIMA1β (EGFP-LIMA1) construct. We also used Gibson assembly to create an N-terminally tagged mScarlet3-LIMA1β (mS3-LIMA1) construct; sequence encoding mScarlet3 was purchased from Addgene (Plasmid # 189753).

### Tagging of endogenous EPS8 with mStayGold

A guide RNA (gRNA) for targeting human EPS8 was generated using the Benchling CRISPR design tool. A single gRNA 20 bp in length with an NGG PAM site was selected based on the on-target score and guide RNA overlap with output from the GenScript gRNA program. gRNA sequences were adapted to include overhangs complementary to the PX459 backbone. Oligos were synthesized by Sigma-Aldrich and resuspended at a concentration of 100 µM. Following the Zhang laboratory Addgene protocols (https://www.addgene.org/crispr/zhang/), the PX459 plasmid was cut using Bbs I and gel purified (Macherey-Nagel #740609.50). Forward and reverse oligos were phosphorylated, annealed, ligated into the cut PX459 vector, which was subsequently used to transform DH5α bacteria. Plasmid DNA from positive clones was purified and sequenced using whole plasmid sequencing. A model repair construct encoding mStaygold plus a linker sequence, flanked by homology arms that target immediately upstream of the *Hs* EPS8 exon 2, was created in silico using the GenScript CRISPR design program. This sequence was used to design 100 bp forward and reverse primers, which contained 80 bp of genomic sequence, and 17 bp or 10 bp of mStayGold sequence, respectively (Forward primer, 5′GAAACTGAGATGTCTTGACCTTTTTATTGCCTTTAATCTATGTGTCTTTCTTTTTTTAGAACACACAAGTGAAAGACACAATGgtgtctacaggcgagga 3′, 80bp EPS8 homology arm, ATG added for start codon, and 17 bp mStayGold homology; Reverse primer, 5′AAACTTTTCAACTTACTTCATCTGAGATGGGTACATTCCAAAACTACTGGGATGATTAGAAATATGACCATTCATAGCGGAGGAGGAGGAcaggtgggcc 3′, 75 bp EPS8 homology arm, 15 bp SGGGG linker, and 10 bp mStayGold homology). A start codon was added to the forward primer and a linker sequence encoding SGGG to the reverse primer. PCR was performed using the Guide It Long ssDNA Strandase Kit V2 (Takara #532667) to generate double-stranded DNA encoding the full repair construct containing the mStayGold sequence. This PCR product was confirmed by gel electrophoresis and subsequently purified (Macherey-Nagel #740609.50). Transfection of gRNA plasmid and double-stranded repair construct DNA into HeLa cells was performed with Lipofectamine 2000 (Thermo Fisher #11668019) in a 6-well format. Cells were left in transfection reactions for 2 d, expanded into T-175 flasks, and then sorted as single cells into 96-well plates by the Vanderbilt Flow Cytometry Core. Positive cells were identified by plating clones onto a glass-bottom 6-well plate and imaging with a spinning disk confocal.

### Transient transfections

Transfections were performed using Lipofectamine 2000 (Thermo Fisher #11668019) according to the manufacturer's protocol. Cells were incubated in Lipofectamine and transfected with 2–4 µg total of DNA for 4–6 h before passaging and replating onto 35 mm #1.5 glass-bottom dishes or coverslips (Cellvis #D35-20-1.5- 23 N). 24 h before fixation, W4 cells were supplemented with 1 mg/ml Doxycycline (RPI; D43020) to induce differentiation. Cells were either fixed or imaged live the following day.

### CellBrite membrane dye

CellBrite SteadyDye650 (Biotium #30108) and CellBrite Steady Enhancer (Biotium #99855) were added to the cell culture media of cells plated on 35 mm #1.5 glass-bottom dishes at 1 µg/ml, 30 min to 1 h before the media was removed and replaced with fresh cell culture media containing 0.5% methylcellulose.

### Frozen mouse tissue preparation

Segments of proximal intestine were removed, flushed with phosphate-buffered saline (PBS), and prefixed for 15 min with 4% paraformaldehyde (PFA) to preserve tissue structure. The intestinal tube was then cut along its length, subdissected into 0.5 mm chunks, fixed for an additional 30 min in 4% PFA at RT, and washed three times in PBS. Whole-mount samples were then moved to Eppendorf tubes for staining. For frozen sections, tissue samples were gently placed in a cold 30% sucrose solution with 1% sodium azide and allowed to sink to the bottom overnight at 4°C. Specimens were then swirled in three separate blocks of Optimal Cutting Temperature compound (OCT; Electron Microscopy Sciences), oriented in a block filled with fresh OCT, and snap frozen in dry ice-cooled acetone. Samples were cut into 10-µm thick sections that were mounted directly on slides for staining.

### Immunofluorescence

***Mouse frozen tissue staining***. Frozen tissue sections were rinsed three times in PBS and permeabilized with 0.1% Triton X-100 in PBS for 15 mins at RT. After permeabilization, tissue slides were rinsed three times in PBS and blocked with 10% BSA (Research Products International #9048-46-8) in PBS for 2 h at 37°C in a humidified chamber. Immunostaining was performed using primary antibodies (LIMA1, Sigma-Aldrich, Catalogue# HPA052645) diluted in 1% bovine serum albumin (BSA) overnight at 4°C. After incubation with primary antibody, slides were rinsed three times in PBS and incubated for 2 h with goat anti-rabbit Alexa Fluor 488 F(ab’)2 fragment (Thermo Fisher Scientific, Catalogue# A-11070) at 1:1000 and an Alexa Fluor-647 conjugated phalloidin (Thermo Fisher Scientific, Catalogue# A22287) at 1:200 at RT. Slides were then washed three times with PBS, and coverslips were mounted with 60 µl ProLong Gold (Invitrogen #P36930) overnight.

***Paraffin-embedded tissue staining***. Human intestinal tissue samples were generously provided by Dr. Izumi Kaji (VUMC). Using a Tissue-Tek II manual slide staining set, slides were deparaffinized in Histo-Clear II (National Diagnostics) twice for 3 min. Tissue was then rehydrated in a descending ethanol series (100%, 100%, 95%, 90%, 70%, and 50%) for 5 min per dilution, followed by washing in PBS three times for 3 min. Slides were boiled in antigen retrieval buffer (10 mM Tris, 0.5 mM EGTA, pH 9.0) in Coplin jars for 1 h using a rice cooker and then cooled to RT. Slides were washed three times, 3 min each in PBS, and then blocked in 10% NGS for 1 h at RT. Primary antibody (LIMA1, Sigma-Aldrich, #HPA052645; Villin, Santa-Cruz Biotechnology, # sc-66022) diluted in 1% NGS was added overnight at 4°C. The next day, slides were washed three times, 5 min each in PBS and goat anti-rabbit Alexa Fluor 488 F(ab’)2 fragment (Thermo Fisher Scientific, Catalogue# A-11070) and goat-anti-mouse Alexa Fluor 647 (Molecular Probes, #A11031) at 1:1000 diluted in 1% NGS was added for 1 h at RT in the dark. Slides were then washed three times, 5 min each in PBS, followed by dehydration with an ascending ethanol series (50%, 70%, 90%, 95%, 100%, and 100%), 5 min each. Coverslips were mounted with ProLong Gold.

***Cultured cell staining***. For fixed sample imaging, cells were washed three times with pre-warmed phosphate-buffered saline (PBS) before being fixed in 4% paraformaldehyde (Electron Microscopy Sciences #15710) in PBS for 15 mins at 37°C. Cells used for fascin antibody staining were fixed with ice-cold methanol and kept at −20°C for 15 min before washing. For antibody staining, cells were permeabilized with Triton X-100 for 10 min at RT and then washed three times in PBS. Primary antibody (LIMA1, Sigma-Aldrich, Catalogue# HPA052645; Fascin, Dako, #M3567; VASP, Cell Signaling, #3132S) was added to coverslips in 1% BSA for 1 h at 37°C and then washed three times with PBS. Cells were then incubated for 1 h with goat anti-rabbit Alexa Fluor 488 F(ab’)2 fragment (Thermo Fisher Scientific, Catalogue# A-11070) at 1:1000 in 1% BSA in addition to Alexa Fluor 647 phalloidin (1:200; Invitrogen #A12380) at RT. Coverslips were then washed three times in PBS and mounted on slides with 20 µl ProLong Gold (Invitrogen #P36930).

### Light microscopy

Mouse frozen intestinal tissue sections were imaged with a Nikon N-SPARC equipped with two excitation LASERS (488 nm and 647 nm) and a Nikon 100X/1.49 Apo TIRF oil immersion objective. Images presented in figures were deconvolved using Richardson-Lucy deconvolution of image volumes, 12 iterations, with Nikon Elements software. Images of paraffin-embedded human intestinal samples were collected on a Zeiss LSM980 Airyscan microscope with four excitation LASERs (488 nm, 568 nm, 647 nm, and 405 nm), and a Zeiss 63X/1.43 NA oil immersion objective; images were processed using Zeiss Zen software. Spinning disk confocal imaging was conducted using a Nikon Ti2 inverted light microscope with a Yokogawa CSU-W1 spinning disk head, a Photometrics Prime95B sCMOS camera, four excitation LASERs (488 nm, 568 nm, 647 nm, and 405 nm), and a Nikon 100X/1.49 NA Apo TIRF oil immersion objective or a Nikon 60X/1.49 NA Apo TIRF oil immersion objective. Images presented in figures labeled as deconvolved were processed using Richardson-Lucy deconvolution with 12 iterations using Nikon Elements software.

### Scanning electron microscopy

Glass coverslips were indexed by placing an alphanumeric pattern mask over the coverslip, followed by platinum sputter coating. HeLa cells were transiently transfected with EGFP-LIMA1 for 4–6 h before plating on indexed glass coverslips in a 6-well dish. After 24 h of plating, cells were washed three times with PBS to remove all media and were then fixed in a buffer of 2.5% glutaraldehyde and 0.1 M CaCl_2_-free cacodylate overnight at RT. Coverslips were washed three times in 0.1 M CaCl_2_-free cacodylate buffer and incubated in pre-warmed 1% tannic acid in cacodylate. Following washes with 0.1 M cacodylate, coverslips were incubated for 1 h in 1% OsO_4_. After washing with ddH_2_O, they were incubated for 30 min with 1% uranyl acetate in ddH_2_O. Finally, coverslips were washed with ddH_2_O three times for 5 min to remove uranyl acetate, before dehydrating with a series of graded EtOH washes. After samples were dried using critical point drying and vented for 20 min, coverslips were mounted on SEM specimen stubs via a conductive adhesive tab and sputter-coated before imaging. SEM imaging was performed on a Zeiss Crossbeam 550 with a voltage of 2 keV and an SE2 detector, using Atlas 5 acquisition software. All reagents were purchased from Electron Microscopy Sciences.

### Correlative light electron microscopy (CLEM)

Glass coverslips were indexed by placing an alphanumeric pattern mask over the coverslip, followed by platinum sputter coating. HeLa cells were transiently transfected with EGFP-LIMA1 for 4–6 h before plating on indexed glass coverslips in a 6-well dish. Before fixation, cells were imaged using 488 epifluorescence on a Nikon Eclipse TS2 cell culture microscope to locate cells with EGFP-LIMA1 and DIC to image their location on the platinum grid. Cells were then fixed, prepared for imaging, and imaged using scanning electron microscopy (refer to the previous section for details). The FIJI plugin Big Warp (BigDataViewer) was used to align EGFP fluorescent images with electron micrographs of the same cell.

### Live imaging and fluorescence recovery after photobleaching (FRAP)

HeLa cells were imaged live using a Nikon Ti2 inverted light microscope equipped with a Yokogawa CSU-X1 spinning disk head, a Photometrics Prime95B sCMOS camera, three excitation LASERs (488 nm, 568 nm, and 647 nm), a Nikon 100X/1.49 NA Apo TIRF oil immersion objective, and a Tokai Hit microincubation stage to maintain environmental control. To reduce filopodial waving, cells were imaged in media containing 0.25% methylcellulose. To perform FRAP analysis, Nikon Elements was used to draw a square ROI in a region along a single filopodium. A stimulating 405 nm LASER controlled by a Bruker mini-scanner set at 70% power and a dwell time of 40 µs was targeted to each ROI. During the acquisition, a single image was acquired before bleaching, and post-bleach fluorescence recovery was captured at 2.5 s intervals for 2 min. For FRAP experiments, HeLa cells were cultured in media containing 0.5% methylcellulose to further dampen lateral filopodial motion.

### Data analysis

All comparative datasets were generated from cells that were overexpressing LIMA1 vs. non-expressing cells in the same sample.

***Linescans of LIMA1 localization in W4 and HeLa cells***. Lines measuring the intensity of phalloidin staining and endogenous LIMA1 antibody or exogenous mS3-LIMA1 were drawn from the base to the tip of microvilli and filopodia in W4 and HeLa cells, respectively. Analyzed images consisted of MaxIPs of limited volumes, which enabled the visualization of complete microvilli or filopodia; five microvilli/filopodia were measured per cell. W4 cells were deconvolved (Richardson-Lucy, 12 iterations) before linescan analysis.

***Microvilli length measurement in W4 cells***. Images containing W4 cells with polarized brush borders were deconvolved (Richardson-Lucy, 12 iterations), and single slices encompassing whole microvilli were used. Measurements were made using hand-drawn lines for 4–10 microvilli per W4.

***Surface localization of microvilli in W4 cells***. Images of W4 cells were resliced and visualized laterally (in the x–z axis); these images were then used to score the position of F-actin densities as lateral or dorsal. Cells were not scored if the phalloidin signal was unpolarized.

***Linescans for LIMA1 localization in CL4 cells***. Images of CL4 cells were deconvolved in Nikon Elements (Richardson-Lucy, 12 iterations), opened in FIJI, and resliced in the x–z axis. MaxIPs of two image planes were used to visualize microvilli. Linescans for phalloidin and mS3-LIMA1 intensity were drawn from the base of the microvillus to the tip with a line width of 4 pixels.

***Microvilli length measurements in CL4 cells***. Images of CL4 cells were deconvolved using Richardson-Lucy for 12 iterations with Nikon Elements software and then projected in 3D. The 3D line length tool was used to measure the length of individual microvilli. Protrusions were selected based on visual clarity, and 10–20 microvilli were measured per cell.

***Distance of EPS8 puncta to HeLa cell body***. SDC images of endo-EPS8 HeLa cells or endo-EPS8 plus mS3-LIMA1 OEx HeLa cells were opened in Nikon Elements software and cropped to the most basal ∼7 *z*-slices. A binary was hand-drawn surrounding all filopodia that were not entangled with neighboring cells. A second binary was drawn around the perimeter of the cell body, excluding filopodia. Both binaries were applied to all *z*-slices. Bright spots detection (diameter: 0.55 µm, contrast: 21.0, intensity: 12-max) was used to locate EPS8 puncta in the filopodial binary area. A child-distance measurement was used to determine the straight-line distance from each EPS8 punctum (child) to the cell perimeter (parent). Averages of straight-line distances per cell were used in the analysis.

***Length of filopodia in HeLa cells***. MaxIPs were generated from up to 10 z-slices, lines were drawn along filopodia selected based on visual clarity, and the lengths of 8–12 filopodia were measured per cell.

***Area Under the Curve (AUC) analysis***. To measure the impact of LIMA1 expression on the thickness of core actin bundles, lines were drawn (5 pixels in width, 1.3 µm in length) orthogonal to individual protrusions visualized in the phalloidin channel, with four to 13 filopodia measured per cell. Linescans were drawn to ensure that lines only crossed through a single protrusion. Areas under the resulting intensity curves (AUC) were then calculated in Prism.

***Number of EPS8 puncta per micron and mean punctum intensity***. SDC images of endo-EPS8 HeLa cells with mS3-LIMA1 overexpression were deconvolved (Richardson-Lucy, 12 iterations), and a line was drawn along segments of the cell perimeter that were visually clear (no contact with neighboring cells, filopodia all contained within the image). A mask was applied that encapsulated all filopodia along the line and excluded the cell body. A bright spots detection binary was applied within the mask to capture EPS8 puncta, and the number of objects was measured per cell. The number of EPS8 puncta was divided by the perimeter line length to calculate the number of EPS8 puncta per micron perimeter. To capture EPS8 intensity, 488 intensity was captured from the thresholded EPS8 puncta, and a mean 16-bit intensity was calculated per cell.

***EPS8 puncta tracking***. Single z-slices of endo-EPS8 HeLa cells with and without mS3-LIMA1 overexpression were imaged for 15 min with images collected every 2.5 s in media containing 0.5% methylcellulose. Areas of the cell with clear, unobstructed filopodia were cropped, and boundaries were drawn to capture EPS8 puncta in filopodia, but not in the cell body. TrackMate, a FIJI plugin, was used to identify the locations of EPS8 puncta with subpixel precision and then track the movement of each punctum over the course of the movie. Tracks were discarded if they lasted less than 30 s.

***Kymograph for EPS8 and β-actin photobleaching***. For filopodia growth rate measurements, culture medium was replaced before imaging with fresh complete DMEM supplemented with 0.5% methylcellulose to reduce protrusion lateral waving and drift. Movies were collected at 2.5 s intervals over 15 min. Kymographs were generated for individual filopodia using Nikon Elements software. Approximately 20–50 filopodia were analyzed per cell, with four cells analyzed per replicate, from three independent biological replicates. Filopodia velocity rates were quantified by measuring the slope of puncta trajectories along the filopodial axis, using the velocity analysis function in Nikon Elements. Instances of positive slopes were counted as filopodial growth events, while negative slopes were counted as filopodial retraction events. Data from individual filopodia were pooled within each biological replicate. β-actin treadmilling rates were quantified using mNeonGreen-β-actin photobleaching (refer to FRAP section), by measuring the slope of fiducial marks from bleached ROIs in kymographs drawn along the filopodial axis, using the velocity analysis function in Nikon Elements. Movies were collected at 2.5 s intervals over 3 min; three to 10 filopodia were measured per cell.

***Fluorescence Recovery After Photobleaching (FRAP) analysis***. A background ROI was used to account for whole-field photobleaching and background fluorescence. Fractional recovery versus time was calculated from the fluorescence intensity by subtracting a background ROI from the fluorescence intensity in the bleached ROI. The data were then normalized, and recovery curves were fit with a one-phase association model in Prism. The immobile fraction was calculated as 1—the fit plateau.

***Persistence of motion and velocity of filopodia in HeLa cells***. 15-min movies of endo-EPS8 cells with and without mS3-LIMA1 overexpression were captured using 0.5% methylcellulose in the media to reduce lateral waving of filopodia. MaxIPs of small, cropped volumes of each cell, which included filopodia that did not leave the frame during the time-lapse, were used for this analysis. TrackMate, a Fiji plugin, was used to track EPS8 puncta with subpixel localization, and a boundary was used to exclude EPS8 puncta in the cell body. Persistence of motion and velocities were derived from the resulting trajectories.

## Supporting information





Supporting Video 1 Movie S1 **LIMA1 overexpression drives the formation of exaggerated and dynamic filopodia in HeLa cells.** MaxIP of 100x SDC timelapse volume acquired at 15 sec intervals over a 30 min period of HeLa cells overexpressing mS3-LIMA1. LUT shows LIMA1 intensity. Scale bar = 10 μm.

Supporting Video 2 Movie S2 **Filopodia in LIMA1 OEx HeLa cells exhibit dynamic merging.** Single z-slice 100x SDC timelapse of an mS3-LIMA1 overexpressing endo-EPS8 HeLa cell imaged at 2.5 sec intervals over a 10 min period.

Supporting Video 3 Movie S3 **Filopodia in LIMA1 OEx HeLa cells exhibit dynamic branching.** Single z-slice 100x SDC timelapse of an endo-EPS8 HeLa cell overexpressing mS3-LIMA1 imaged at 2.5 sec intervals over a 30 min period.

Supporting Video 4 Movie S4 **Filopodia in LIMA1 OEx HeLa cells exhibit dynamic merging.** Single z-slice 100x SDC timelapse of an mS3-LIMA1 overexpressing endo-EPS8 HeLa cell imaged at 2.5 sec intervals over a 10 min period. CellBrite was used to mark the membrane.

## Abbreviations used:

AUC: area under the curve
CLEM: correlative light electron microscopy
CL4: LLC-PK1-CL4
EGFP: enhanced green fluorescent protein
EPS8: epidermal growth factor receptor pathway substrate 8
FFPE: formalin fixed and paraffin embedded
FRAP: fluorescence recovery after photobleaching
LIMA1: LIM-domain and Actin Binding 1
mNG: mNeonGreen
MISP: mitotic spindle positioning
mS3: mScarlet3
NSPARC: Nikon spatial array confocal
SEM: scanning electron microscopy
W4: Ls174T-W4.
