# Supplementary material for: A role for LIMA1 in the assembly of actin bundle-supported protrusions
Source: Mol Biol Cell. 2026 Jun 10;37(7):ar66. doi: 10.1091/mbc.E26-02-0073 (PMC13329885; doi:10.1091/mbc.E26-02-0073)
Supplement: Supplementary file 1 [file mbc-37-ar66-s001.pdf]

Supplemental Materials

Molecular Biology of the Cell

Silverman *et al*.

SUPPLEMENTAL INFORMATION

“A role for LIMA1 in the assembly of actin bundle-supported protrusions”

By Silverman et al.

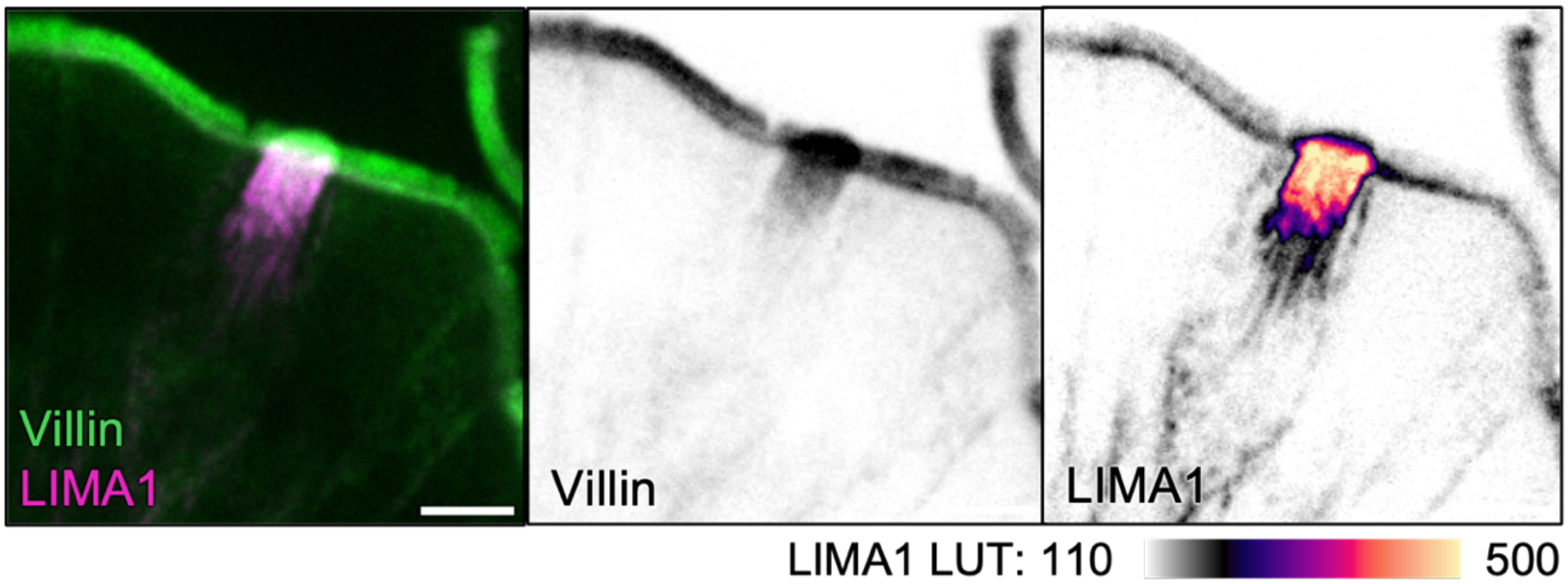

Figure S1. LIMA1 localizes to the rootlets of microvilli in mouse intestinal tissue. Single slice 100x SDC image of formalin fixed and paraffin embedded mouse intestinal tissue showing antibody staining for LIMA1 and Villin. LIMA1 antibody staining marked with intensity-scaled LUT. Scale bar = 5 μm.

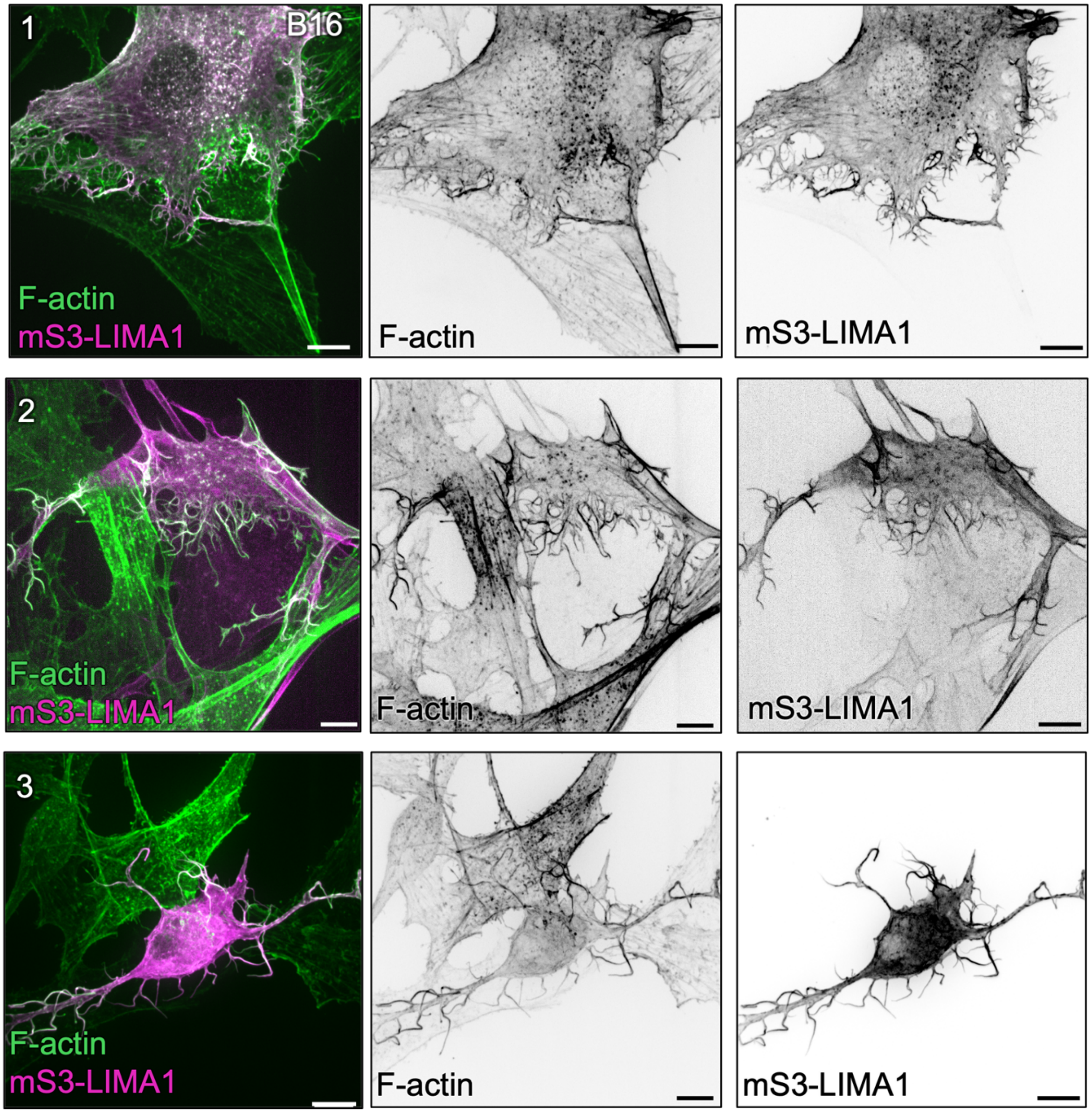

Figure S2. LIMA1 overexpression drives exaggerated filopodia formation in mouse B16 cells. MaxIPs of 100x SDC image volumes showing three different examples of B16 cells overexpressing mS3-LIMA1 (magenta) and phalloidin (green). Scale bar = 10 μm.

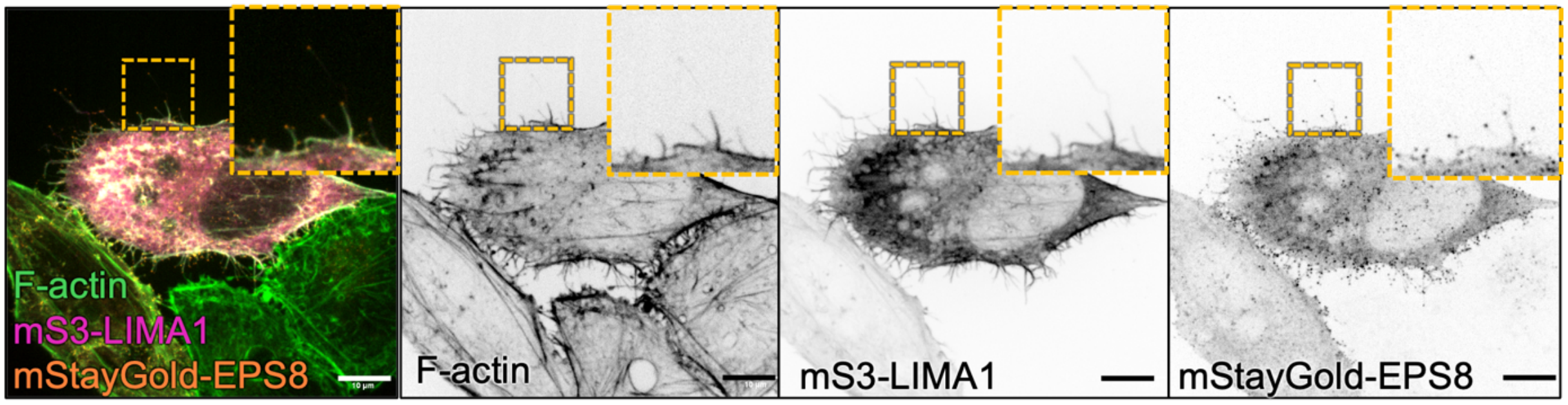

Figure S3. Overexpressed mStayGold-EPS8 localizes to the tips of filopodia. MaxIP of 100x SDC image volume showing HeLa cells overexpressing mS3-LIMA1 (magenta) and mStayGold-EPS8 (orange). Scale bar = 10 μm.

Table S1 - FRAP curve fitting parameters

One-phase association fit	EGFP-LIMA1	EGFP-ESPIN	EGFP-FIMBRIN	EGFP-MISP
**Best-fit values**				
Y0	0.0371	0.008241	0.02374	0.01917
Plateau (mobile fraction)	0.5315	0.2232	0.9285	0.3477
Immobile fraction	0.4685	0.7768	0.0715	0.6523
K	0.03005	0.03824	0.01704	0.03222
Tau	33.28	26.15	58.67	31.03
Half-time	23.07	18.12	40.67	21.51
**95% CI (likelihood)**				
Y0	0.01306 to 0.06057	-0.004739 to 0.02088	-0.001551 to 0.04859	-0.003117 to 0.04076
Plateau (mobile fraction)	0.5092 to 0.5593	0.2150 to 0.2329	0.8637 to 1.017	0.3299 to 0.3714
K	0.02544 to 0.03492	0.03247 to 0.04446	0.01416 to 0.02000	0.02582 to 0.03914
Tau	28.64 to 39.31	22.49 to 30.80	50.00 to 70.64	25.55 to 38.74
Half-time	19.85 to 27.25	15.59 to 21.35	34.66 to 48.97	17.71 to 26.85
**Goodness of Fit**				
Degrees of Freedom	2677	1837	1837	2037
R squared	0.389	0.3976	0.6264	0.3125
Sum of Squares	73	9.226	49.86	34.67
Sy.x	0.1651	0.07087	0.1648	0.1305
**Number of points**				
# of X values	3200	3200	3200	3200
# Y values analyzed	2680	1840	1840	2040
